# Epidemiology of Acute Gastroenteritis Outbreaks Caused by Human Calicivirus (Norovirus and Sapovirus) in Catalonia: A Two Year Prospective Study, 2010-2011

**DOI:** 10.1371/journal.pone.0152503

**Published:** 2016-04-27

**Authors:** Nuria Torner, Ana Martinez, Sonia Broner, Antonio Moreno, Neus Camps, Angela Domínguez, Working Group for the Study of Acute Viral Gastroenteritis Outbreaks in Catalonia

**Affiliations:** 1 Public Health Agency of Catalonia, Barcelona, Spain; 2 CIBER Epidemiología y Salud Pública CIBERESP, Carlos III Health Institute, Madrid, Spain; 3 Public Health Department, University of Barcelona, Barcelona, Spain; 4 Public Health Agency of Barcelona, Barcelona, Spain; University of Hong Kong, HONG KONG

## Abstract

**Background:**

The epidemiology of cases of acute gastroenteritis (AGE) of viral etiology is a relevant public health issue. Due to underreporting, the study of outbreaks is an accepted approach to investigate their epidemiology. The objective of this study was to investigate the epidemiological characteristics of AGE outbreaks due to norovirus (NoV) and sapovirus (SV) in Catalonia.

**Material and Methods:**

Prospective study of AGE outbreaks of possible viral etiology notified during two years in Catalonia. NoV and SV were detected by real time reverse transcription polymerase (RT-PCR).

**Results:**

A total of 101 outbreaks were registered affecting a total of 2756 persons and 12 hospitalizations (hospitalization rate: 0.8x1,000,000 persons-year); 49.5% of outbreaks were foodborne, 45.5% person to person and 5% waterborne. The distribution of outbreaks according to the setting showed a predominance of catering services (39.6%), nursing homes and long term care facilities (26.8%) and schools (11.9%). The median number of cases per outbreak was 17 (range 2–191). The total Incidence rate (IR) was 18.3 per 100,000 persons-years (95%CI: 17.6–19.0). The highest IR was in persons aged ≥65 years (43.6x100,000 (95% CI: 41.0–46.2)) (p<0.001). A total of 1065 samples were analyzed with a positivity rate of 60.8%. 98% of positive samples were NoV (GII 56.3%; GI 4.2%; GII+GI 4.2%; non- typable 33.0%). SV was identified in two person-to-person transmission outbreaks in children.

**Conclusions:**

These results confirm the relevance of viral AGE outbreaks, both foodborne and person-to-person, especially in institutionalized persons. SV should be taken into account when investigating viral AGE outbreaks.

## Introduction

Caliciviruses are 27 to 40 nm, nonenveloped, single-stranded RNA viruses of the family *Caliciviridae* with two genus, *Norovirus* and *Sapovirus*, associated with disease in humans [1]. Human caliciviruses (HuCVs) have a worldwide distribution, with multiple antigenic types circulating simultaneously in the same region. Norovirus (NoV) is the most common cause of sporadic viral acute gastroenteritis (AGE) as well as gastroenteritis outbreaks. Because of high levels of contact and vulnerable patient populations, healthcare settings can be particularly susceptible to outbreaks of HuCVs. The most commonly detected HuCVs are noroviruses. There are currently five genogroups (G) of NoVs with strains that infect humans found mainly in GI and GII, and to a much lesser extent in GIV [2] being the NoV genogroup 2 type 4 (GII.4) the predominant during the past decades in the Americas, Europe and Oceania [3–8]. GII.4 strains are more commonly associated with person-to-person transmission, while GI strains are identified more frequently in shellfish-associated outbreaks.

The primary route of transmission of noroviruses is fecal-oral, although airborne transmission also occurs. Contaminations of food, water or fomites and direct person to person spread have all been implicated in outbreaks of NoV gastroenteritis. The incubation period ranges from 10 to 51 hours, and the infectious dose is low. As many as one third of persons shed the virus before the onset of illness, and peak fecal virus shedding may occur after gastroenteritis symptoms have resolved [2].

NoV-associated gastroenteritis occurs in a variety of settings. NoVs are most frequently recognized as causes of outbreaks of acute gastroenteritis, but sporadic illness is also common. NoVs are responsible for 47–96% of outbreaks of acute gastroenteritis and 5–36% of sporadic cases of acute gastroenteritis worldwide [9].

Secondary transmission of NoV infection is common (often >30%), allowing the spread of an outbreak, particularly in closed settings such as healthcare institutions (e.g., hospitals and nursing homes) and cruise ships [10]. The emergence of new variants of the GII.4 and GII.2 strains has been observed but it is not yet clear whether antigenic drift driven by population immunity occurs among other NoV genotypes [11].

NoV infection is usually a self-limited illness, and healthy persons typically recover without sequelae. Elderly and chronically illpersons are more likely to suffer these complications, and death has complicated norovirus outbreaks among elderly residents of nursing home facilities [2]. Sapovirus was first identified in an outbreak of diarrhea in an orphanage in Sapporo, Japan, in October 1977 [12], and since then, 15 genotypes in four genogroups (GI.1 to GI.8, GII.1 to GII.5, GIV, and GV) have been described as human SVs. Sapoviruses have been detected fom a variety of epidemiological sources, including fecal specimens from symptomatic and asymptomatic individuals, environmental water, and bivalves in Japan, indicating that SVs can be transmitted via the fecal-oral route through water and contaminated foods, as well as through person-to-person contact [13;14].

Sapovirus (SV) cause AGE in humans of all ages as sporadic cases although they have increasingly been recognized as a cause of outbreaks. An increasing prevalence of sapovirus infections has been described, highlighting the emerging role of sapoviruses as a public health problem [15–18]. In general, the severity of SV AGE is milder than NoV AGE and the symptoms are self-limiting although, depending on the individual (immunocompromised, small children or elderly people) SV AGE may lead to hospitalization [17;19;20].

There are some clinical differences between NoV and SV. Diarrhea is the most frequent symptom in SV outbreaks where in NoV outbreaks is vomiting and low grade fever [20].

Outbreaks with high attack rates tend to occur in closed populations, such as nursing homes, child care centres, and cruise ships [21]. Transmission is person-to-person spread via the fecal-oral route or through contaminated food or water. Norovirus is the most common cause of foodborne illness and foodborne disease outbreaks in the United States [22;23]. Exposure to contaminated surfaces and aerosolized vomits has been implicated in some outbreaks [24]. Viral excretion peaks 4 days after exposure and may persist for as long as 3 weeks. Prolonged excretion can occur in immunocompromised hosts. Infection occurs year round but is more common during the colder months [25]. The incubation period is 12 to 48 hours. HuCVs are extremely contagious, large numbers of virus particles can be excreted, and shedding can last for several weeks after symptoms have subsided. The spread of infection can be reduced by standard measures for the control of diarrhea, such as educating child care providers and food handlers about infection control, maintaining the cleanliness of surfaces and food preparation areas, using appropriate disinfectants, excluding caregivers or food handlers who are ill or for a period after recovery (e.g., 24–72 hours), exercising appropriate hand hygiene, and excluding children from group child care. Sporadic cases of HuCV are not notifiable nationally, but outbreaks of HuCV should be reported to local and state public health authorities [26].

he lack of accurate diagnostic assays available in clinical laboratory settings results in disease incidence for NoV and other viral causes of AGE being underestimated [22]. With the exception of an enzyme immunoassay (EIA) for rotavirus, the diagnosis of viral AGE is made largely on the basis of clinical signs and symptoms. Molecular techniques used for the definitive diagnosis, specifically polymerase chain reaction (PCR) tests, are mostly only availablein public health laboratories and research settings. Evaluation of viral AGE incidence is further limited by the fact that most AGE patients do not seek medical care, and, of those who do, <20% submit fecal specimens for diagnostic tests [27].

The aim of this study was to describe epidemiological features of acute gastroenteritis outbreaks caused by norovirus and sapovirus in Catalonia i a two-year prospective study.

## Materials and Methods

### Study Design

The study was designed as an epidemiological descriptive study of AGE outbreaks reported in Catalonia, an authonomous region in the northeast of Spain with nearly 7.600.000 inhabitants in 2011 [28] from January 1st 2010 to December 31st 2011. The following definitions were used:

AGE was defined as diarrheal disease of rapid onset presenting together with nausea, vomiting, fever, or abdominal pain. A confirmed case of AGC was defined as a patient with ≥2 loose stools and/or ≥2 episodes of vomiting within 24 h, with detection of sapovirus or norovirus in faeces.Outbreaks of AGE were defined as AGE affecting ≥2persons with the same exposure from a common source of infection or by person-to-person transmission. Reporting of AGE outbreaks to the Public Health Agency of Catalonia is mandatory. Survey data was gathered from all suspected viral AGE outbreaks reported to thecorresponding public health surveillance units during the study period. The mode of transmission was ascertained by epidemiological investigation. For each case, sociodemographic data and information about type and duration of symptoms and healthcare received were collected. Patients were asked about AGE symptoms, diarrhea and vomiting, and additional symptoms such as nausea, fever, abdominal pain, headache, myalgia, malaise and chills. Information was collected by staff of the epidemiological research units using a standardized questionnaire. Stool samples were collected from patients, healthcare or daycare staff and foodhandlers by public health epidemiologists as part of the outbreak control procedures. Initial information identifying patients was anonymized prior to analysis.

### Laboratory Procedure

Stool samples were pre-screened using standard microbiological tests to rule out bacteria and parasites and stored at −20°C before testing by enzyme immunoassay (EIA).

Stool samples were plated on selective and differential media to study *Salmonella* (MacConkey agar, Salmonella-Shigella agar, Xylose-Lysine-Desoxycholate agar and Selenite enrichment broth), *Shigella* (MacConkey agar and Salmonella-Shigella agar), Shiga toxin-producing strains of O157:H7 *Escherichia coli* (MacConkey agar with sorbitol), *Yersinia* (Cefsulodin-Irgasan-Novobiocin, CIN agar), *Campylobacter* (Charcoal agar), *Vibrio* (Thiosulfate Citrate Bile salt Sucrose, TCBS agar) and *Aeromonas* spp (Pseudomonas-Aeromonas agar with 100,000 IU per litre of Penicillin G, GSP agar). In outbreaks where a parasitic infection was suspected, the diagnosis was established by direct microscopic examination or after concentration of preserved stool (Merthiolate- iodine-formalin and 10% formalin) to determine the presence of ova, trophozoites or cysts. *Cryptosporidium* oocysts were examined by stained fecal materials (Auramine and Ziehl-Neelsen stains).

Additionally, reverse transcriptase-polymerase chain reaction (RT-PCR) detection was performed. Specimens underwent molecular testing for norovirus, sapovirus, astrovirus and enteric adenovirus at the laboratory (Universitari H. Vall d’Hebron Microbiology Laboratory, Public Health Agency of Barcelona Laboratory and Enteric Virus Laboratory, University of Barcelona) Viral nucleic acid was extracted and TaqMan RT PCR and PCR were used for initial sample screening for genogroup I (GI) and genogroup II (GII) NoV and SV, and to determine the genotype and viral load of NoV and SV [29;30]. Sequencing, phylogenetic analysis, and genotype assignment PCR products were purified and sequenced with the ABI PRISM BigDye^®^Terminator Cycle Sequencing Ready Reaction Kit V3.1 [4]. For each outbreak, all stool samples were investigated if there were fewer than 10 cases, and at least 10 samples were studied in outbreaks with more than 10 cases. Faecal samples were collected during the acute phase (first 3–5 days) of infection: a minimum of 1 g of stool was collected in a sterile, plastic container without preservative. In outbreaks where less than three samples were collected, Kaplan’s criteria were followed [31].

Outbreaks of suspected viral aetiology that were negative for NoV were analysed for SV using quantitative real-time PCR with primers as previously described [29]. NoV genogroup assignment was performed after amplification by semi-nested RT-PCR of the ORF1/ORF2 junction region (region C) as previously described [4]. Sapovirus genogroup assignment was performed after nested RT-PCR amplification and sequencing [14].

### Statistical Analysis

The following data were collected for each outbreak were studied: setting, date of onset of first case, number of people exposed and affected, age, gender, use of health services, hospitalization and work or school absenteeism, as well as occupation within outbreak setting and laboratory stool testing results. The incidence rates of AGE cases and outbreaks and their 95% confidence intervals (CI) assuming a Poisson distribution were calculated using the estimated population of Catalonia (Statistical Institute of Catalonia (IDESCAT) for 2010 and 2011. Non-normal variables were stated as mediana and ranges. The Mann–Whitney U-test was used to assess the equality of medians for two observation samples. The level of statistical significance was established as *α* = 0.05. The odds ratios (OR) and corresponding 95%CI were calculated to estimate the association between clinical symptoms and age. All statistical analyses were performed by using IBM SPSS^®^ version 18 statistical program (SPSS Inc., Chicago, IL, USA) and R 2.13.0 (R Development Core Team 2011) program.

No ethics committee approval was needed and written consent was not obtained because data were anonymized prior to analysis and gathered for outbreak control and public health purposes.

## Results

During the two year study period, 286 AGE outbreaks were reported, of these 35.3% were of viral etiology, 13% caused by *Salmonella spp* and 20% were of unknown etiology.

A total of 101 (35.3%) viral AGE outbreaks were registered (66 in 2010; outbreak rate: 8.8x1,000,000 persons-year) and 35 in 2011 (outbreak rate: 4.6x1,000,000) affecting 2756 persons with 12 hospitalizations, a hospitalization rate (HR) of 0.8 x1,000,000 persons-year; and 1 death. Of the outbreaks, 49.5% were foodborne, 45.5% person-to-person and 5% waterborne. The distribution of outbreaks according to the setting wasf catering services (39.6%), nursing homes and long-term care facilities (26.8%), and schools (11.9%) (Table 1). The median number of cases per outbreak was 17 (range 2–191). The total incidence rate (IR) was 18.3 per 100,000 persons-years (95%CI:17.6–19.0). By age group, the highest IR was in the ≥65years age group (43.6x100,000 (95%CI 41.0–46.2)) followed by the 0–14 years (17.2x100,000 (95% CI:15.6–19.0)) and 15–64 years (9.2x100,000 (95%CI:8.6–9.8)) age groups (p<0.001). The overall attack rate was 44%, a significant difference in the attack rate was observed between foodborne outbreaks (53.8%) and person-to-person outbreaks (30%) (p<0.001). Both the IR and the risk ratio (RR) for infection were higher in the ≥65 years age group (RR 4.75 (95%CI: 4.35–5.19)) followed by the 0–14 years age group (Table 2). Analysis of the IR by age group and setting showed the highest IR was in the ≥65 years age group and in the catering services setting with 491 affected persons (IR 19.6 x100,000 (95%CI:17.9–21.4)) and in the nursing home setting with 371 affected persons (IR 14.8 x100,000 (95%CI:13.3–16.4)) followed by the 0–14 years age group in the school setting with 245 cases (IR 10.6 x100,000 (95%CI: 9.3–12.0)).

**Table 1:** Distribution of outbreaks according to setting of occurrence and mode transmission. Catalonia 2010–2011.

			Mode of transmission								
			Foodborne		Waterborne		Person to person				
Setting	Number of outbreaks (%)	Persons affected (%)	Number of outbreaks	Persons affected	Number of outbreaks	Persons affected	Number of outbreaks	Persons affected	Persons affected Median(range)	Persons exposed Median(range)	Median age (range)
Household	11(10.9)	84(3)	6a	45	1	15	4	24	6 (2–15)	11 (2–30)	29 (0–82)
Foodservice	40(39.6)	1257(45.6)	35b(2+)	869	-	-	5	388	17 (3–191)	46 (4–3474)	56 (0–93)
School	12(11.9)	436(15.8)	3c	75	1	103	8	258	19 (6–103)	71 (25–488)	10 (0–74)
Holiday camp/ cottage	4(3.9)	53(1.9)	1d	7	1+	17	2	29	13 (7–20)	60 (13–77)	10 (8–55)
Nursing home	14(13.9)	445(16.2)	4e	122	-	-	10	323	28 (12–65)	85 (43–213)	81 (21–102)
Longterm care/ hospital	13(12.9)	371(13.5)	1f	48	-	-	12	323	23 (6–76)	57 (20–203)	76 (19–100)
Other*	7(6.9)	110(4.0)	-	-	2	33	5	77	12 (6–28)	43 (26–83)	37 (0–91)
Total	101 (100%)	2756 (100%)	50 (49.5%)	1166 (42.3%)	5 (5%)	168 (6.1%)	46 (45.6%)	1422 (51.6%)	17 (2–191)	50 (2–3474)	51 (0–102))

* Closed institutions (prison, youth fostering center, mentally disabled facility), free camp site, fun run.

^+^ Positive sample to NoV

^a^ 1 oysters, 1 cake, 4 unknown

^b^ 3 clams, 1 durum, 1 pork roast, 2(1+) mussels, 5(1+) oysters, 22 unknown

^c^ 1 sandwich, 2 unknown

^d^ 1 unknown

^e^ 4 unknown

^f^ 1 unknown

**Table 2:** Incidence rates and risk ratio of infection, hospitalization rates, medical consultation and absenteeism according to age groups and gender. Catalonia 2010–2011.

Age group*	IR x 100,000 persons (95% CI)	RR (95%CI)	p	Medical consultation n(%)	Hospitalization n(%)	Absenteeism school/work n(%)
0 to 14 years	17.19 (15.55–18.97)	1.87 (1.66–2.11)	< 0.001	77 (19.3)	3 (0.76)	133 (33.4)
15 to 64 years	9.18 (8.60–9.78)	1		209 (22.3)	1 (0.11)	137 (14.6)
≥ 65 years	43.56 (41.01–46.22)	4.75 (4.35–5.19)	< 0.001	143 (13. 1)	8 (0.73)	3 (0.3)
Gender**						
Men	13.69 (12.87–14.56)	1		198 (19.4)	6 (0.60)	125 (12.2)
Women	19.91 (18.92–20.94)	1.45 (1.34–1.58)	< 0.001	242 (16.0)	6 (0.40)	150 (9.9)

* 219 of the 2756 affected persons were not surveyed

** 109 out of 2537 surveyed lacked information on age and 4 on gender

The largest number of medical consultations was in the 15–64 years age group (209 cases, 2.3%), while the highest hospitalization rate and absenteeism was in the 0–14 years age group with 3 (0.8%) hospitalizations and 133 cases missing school (33.4%) (Table 2).

### Laboratory findings

A total of 1065 samples were analyzed; 702 (65.9%) from cases and 343 (32.2%) from foodhandlers and care givers [20 samples (1.9%) were of unknown origin]. Only two foodborne outbreaks had no samples from cases, although foodhandler samples were available. The positivity rate was 60.8% (648/1065); in 98% of outbreaks with positive samples NoV was identified as the causative agent with the following genogroup distribution: GII56.3%; GI 4.2%; GII+GI 4.2%; non typable 33.3%; GII+GI+*Salmonella* 1%. Forty seven outbreaks (46.5%) were genotyped, being GII.4 the most prevalent genotype (61.7%). SV was identified in two outbreaks of person-to-person transmission in children and one outbreak of foodborne transmission with co- infection with NoV GII. No samples tested positive forr astrovirus nor adenovirus. There was no statistical difference in proportions between genogroup I and II in foodborne outbreaks (9.4% caused by GI and 90.6% by GII) and in person-to-person transmission (10% GI and 90% GII) [OR = 0.94(95% CI: 0.20–4.55) p = 0.79] The largest percentage of samplescollected and of positivity were in the foodservice setting with 33.9% of samples collected and 32.9% of NoV positivity, followed by the longterm/ hospital and nursing home settings with 23.2% and 21.5% of samples collected and 22.8% and 23.5% NoV positivity respectively. Other settings such as schools, household, closed institutions (prison, youth fostering center, mentally disabled facility) and holiday camps had lower sample collection and positivity rates (10%, 4%, 4.8% and 2.6% of outbreak samples collected; 10.6%, 3.5%, 3.2% and 3.4% positivity rates, respectively).

**Fig 1:**
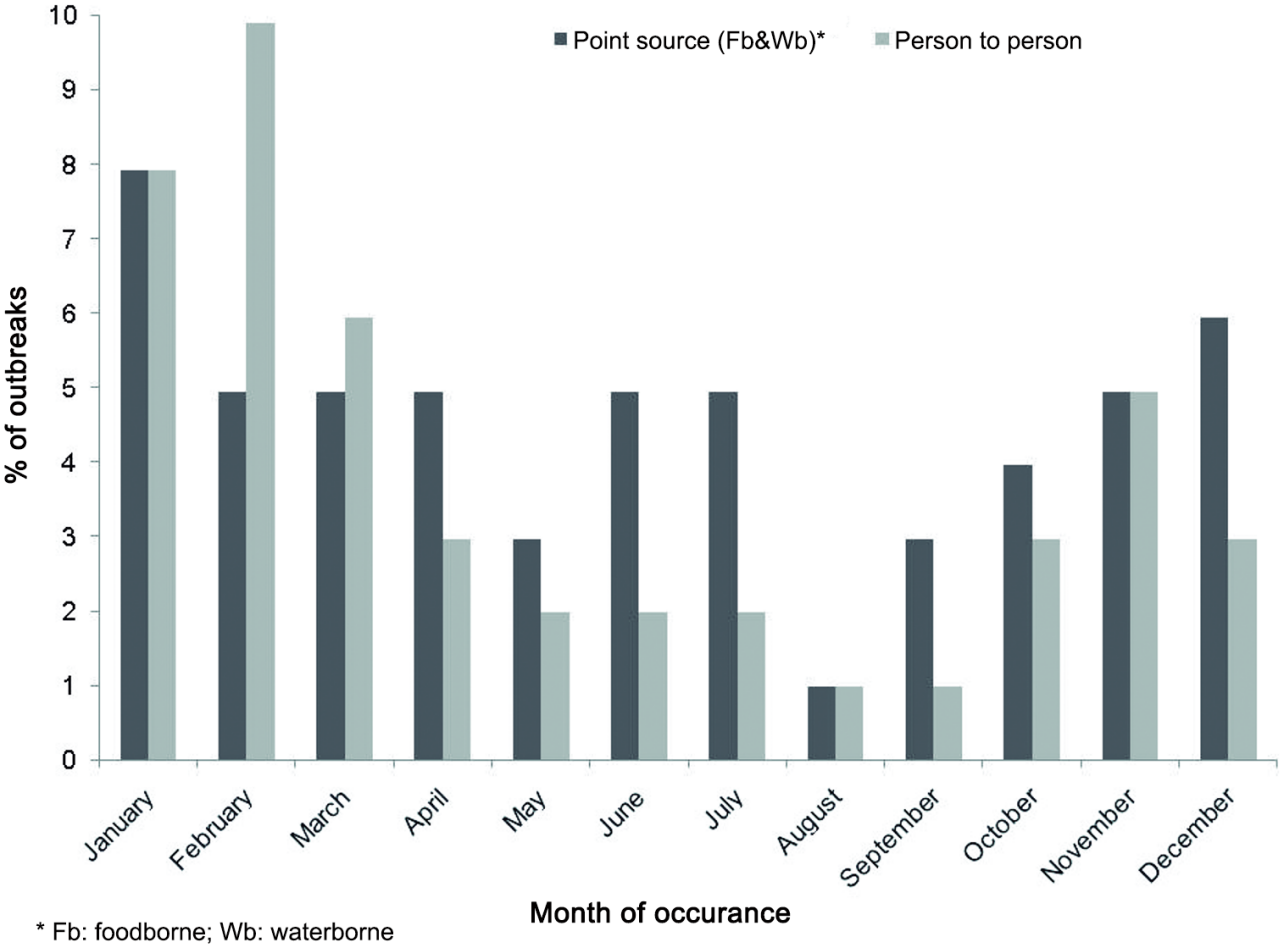
Distribution of acute gastroenteritis outbreaks by month of occurrence and transmission mode. Catalonia 2010 and 2011.* Cold months (November to April) significantly higher incidence than warm months (May to October) for both transmission modes (Table 3).

As to seasonality (Fig 1), there was a significantly higher incidence of viral AGE outbreaks in the cold months (November to April), both for foodborne (38.2% vs. 61.8%) and person-to-person (23.9% vs. 76.1%) outbreaks. Although the warm months had a higher proportion of point source outbreaks than person-to-person outbreaks no significant difference was observed (OR:1.97 (95%CI: 0.82–4.69) p = 0.12)) (Table 3).

**Table 3:** Distribution of percentage of outbreaks by season of the year and transmission mode. Catalonia 2010–2011.

**Season**	**Transmission mode**					
	Common Source (Fb&Wb)*		**Person-to-person**		**Total**	
	n	%	n	%	n	%
Warm (May-Oct)	21	38.2	11	23.9	32	31.7
Cold (Nov-Abr)	34	61.8	35	76.1	69	68.3
p value		0.022		< 0.001		< 0.001
	**Season**					
**Transmission mode**	Warm (May-Oct) Number of outbreaks	Cold (Nov-Abr) Number of outbreaks	OR (95% CI)	p value	**Transmission mode**	Warm (May-Oct) Number of outbreaks
Point source (Fb&Wb)*	21	34	1.97 (0.82–4.69)	0.124	Point source (Fb&Wb)*	21
Person-to-person	11	35	-	-	Person-to-person	11
Total	32	69			Total	32

* Fb: foodborne; Wb: waterborne

## Discussion

The use and application of public health data in descriptive studies can be useful despite underreporting., even in diseases with a high frequency [32]. One aim of this study was to assess the relevance of SV as a causative agent of AGE. The results of this study, partially presented by Sala et al., are the first to report the involvement os SV in three outbreaks, affecting children (2 outbreaks) and adults (1 outbreak) in Catalonia [20]. An environmental study carried out in Catalonia by Sano et al. suggests the emergence of SVs as human pathogens with high environmental prevalence [12]. Svraka et al. described changes in SV epidemiology in the Netherlands, with outbreaks and infections in people aged >60 years, which might suggest the prevalence of SV infection, may be increasing [15]. A thorough literature review [33] also supports the findings of this study as to the evident rise in importance of calicivirus, especially NoV, as causes of illness.

Human norovirus (HuNoV) is a well studied cause of foodborne disease outbreaks worldwide and high-risk foods for HuNoV contamination include shellfish, fresh produce, and commercially prepared uncooked foods [34]. There was higher proportion of outbreaks with a point source transmission (49.5% foodborne and 5% waterborne) than person-to-person (44.8%) transmission, although the directly implicated food was positive in only 4 outbreaks, 3 foodborne in shellfish-related outbreaks in the foodservice setting and one waterborne.

Foodborne and waterborne outbreaks were associated with multiple strains (GI+GII). Waterborne outbreaks were significantly associated with GI strains, while healthcare-related and winter outbreaks were associated with GII strains in accordance with other published outbreak summaries [35].

Both Incidence rate and the risk ratio (RR) for infection were higher in the ≥65years age group followed by the 0–14 years age group. This supports previous findings in Catalonia and in other studies carried out in Spain and other countries [5;33;35–38]. The high hospitalization rate could also be explained by the higher prevalence of GII.4 and a higher degree of severity affecting mainly elderly patients [39]. As described by other authors, our results suggest that the elderly are highly vulnerable and indicate the need to introduce simple tests for an early identification of NoV in cases of AGE affecting the elderly in order to improve care by reducing unnecessary treatments and hospital stays [36].

The seasonality of pathogens is defined as the appearance of recurrent epidemics at defined periods of the year [40]. Our results confirmed the typical winter seasonality of calicivirus with a higher incidence of viral AGE outbreaks from November to April, both for foodborne (38.2% vs. 61.8%) and person-to-person (23.9% vs. 76.1%) transmission modes, although the warm months presented a somewhat higher proportion of point source outbreaks (foodborne and waterborne) than person-to-person transmitted outbreaks. NoV epidemic characteristics, and timing, are remarkably consistent from year to year, with a peak incidence from November to April and specific peaks in February and March [10]. However, NoV outbreaks do occur during the summer. In a previous in Catalonia during 2004–2005 no seasonality was observed, mainly due to the small number of outbreaks during the study period, although higher prevalence of foodborne outbreaks did occur during the warmer months [5]. The seasonal behavior of NoV gastroenteritis is known to be influenced by several hosts and setting factors that can affect attack rates and environmental persistence of the virus.Understanding the seasonal changes in norovirus infection is important to be able to implement efficient surveillance and preventive measures for its control [41].

Our study has some limitations. Using community-based cohort studies would give a more correct estimate of AGE caused by HuCVs and their incidence rates [42] although the study of outbreaks is an accepted approach to investigate the epidemiology of cases of AGE of viral etiology [15;16]. Likewise, some outbreaks may not have been reported and the IR could have been underestimated. Nevertheless, if the underreporting was not differential this should not affect the main findings in each group of outbreaks. Although Kaplan’s criteria were used to confirm the viral etiology in some outbreaks when no samples were available this should not make any difference to the overall study results because it was used in only a few outbreaks.

In conclusion, the general perception is that NoV gastroenteritis is a self-limiting mild illness that rarely requires medical attention, despite several reports of serious illness and death in various settings [43–45]. This study highlights the importance of routine control measures of viral AGE outbreaks in order to reduce school and work absenteeism and hospitalization of elderly and frail cases.

## Supporting Information

S1 Table Distribution and features of NoV&SV outbreaks. Catalonia, 2010–2011.
(RAR)

S2 Table Distribution of cases, foodhandlers and caregivers in NoV&SV outbreaks. Catalonia, 2010–2011.
(RAR)
